# The national rate of intensive care units-acquired infections, one-year retrospective study in Iran

**DOI:** 10.1186/s12889-021-10639-6

**Published:** 2021-03-29

**Authors:** Neda Izadi, Babak Eshrati, Yadollah Mehrabi, Korosh Etemad, Seyed-Saeed Hashemi-Nazari

**Affiliations:** 1 Department of Epidemiology, School of Public Health and Safety, Shahid Beheshti University of Medical Sciences, Tehran, Iran; 2 Department of Social Medicine, School of Medicine, Iran University of Medical Sciences, Tehran, Iran; 3 Prevention of Cardiovascular Disease Research Center, Department of Epidemiology, School of Public Health and Safety, Shahid Beheshti University of Medical Sciences, Tehran, Iran

**Keywords:** Hospital-acquired infections, Rate, Incidence, Intensive care units, Iran

## Abstract

**Background:**

Hospital-acquired infections (HAIs) in intensive care units (ICUs) are among the avoidable morbidity and mortality causes. This study aimed at investigating the rate of ICU-acquired infections (ICU-AIs) in Iran.

**Methods:**

For the purpose of this multi-center study, the rate of ICU-AIs calculated based on the data collected through Iranian nosocomial infections surveillance system and hospital information system. The data expanded based on 12 months of the year (13,632 records in terms of “hospital-ward-month”), and then, the last observation carried forward method was used to replace the missing data.

**Results:**

The mean (standard deviation) age of 52,276 patients with HAIs in the ICUs was 47.37 (30.78) years. The overall rate of ICU-AIs was 96.61 per 1000 patients and 16.82 per 1000 patient-days in Iran’s hospitals. The three main HAIs in the general ICUs were ventilator-associated events (VAE), urinary tract infection (UTI), and pneumonia events & lower respiratory tract infection (PNEU & LRI) infections. The three main HAIs in the internal and surgical ICUs were VAE, UTI, and bloodstream infections/surgical site infections (BSI/SSI). The most prevalent HAIs were BSI, PNEU & LRI and eye, ear, nose, throat, or mouth (EENT) infections in the neonatal ICU and PNEU & LRI, VAE, and BSI in the PICU. Device, catheter, and ventilator-associated infections accounted for 60.96, 18.56, and 39.83% of ICU-AIs, respectively. The ventilator-associated infection rate was 26.29 per 1000 ventilator-days. Based on the Pabon Lasso model, the lowest rates of ICU-AIs (66.95 per 1000 patients and 15.19 patient-days) observed in zone III, the efficient area.

**Conclusions:**

HAIs are common in the internal ICU wards. In fact, VAE and ventilator-related infections are more prevalent in Iran. HAIs in the ICUs leads to an increased risk of ICU-related mortality. Therefore, to reduce ICU-AIs, the specific and trained personnel must be responsible for the use of the devices (catheter use and ventilators), avoid over use of catheterization when possible, and remove catheters earlier.

**Supplementary Information:**

The online version contains supplementary material available at 10.1186/s12889-021-10639-6.

## Introduction

Hospital-acquired infections (HAIs) in intensive care units (ICUs) are among the avoidable causes of morbidity and mortality, which also lead to increased hospital length of stay (LOS), associated costs, and multiple antibiotic resistance, especially in developing countries [1].

These infections are usually acquired after hospitalization and manifest 48 h after admission to the hospital HAIs define as a infections acquired after admission and occur within 48–72 h after admission to the hospital or up to 6 weeks after discharge [2]*.* In addition, HAIs by staff or newborn infections occurring during childbirth are also defined as HAIs [3].

Patients in ICUs are twice as likely to face a HAIs than patients in the general wards [4]. The global prevalence of ICU-acquired infections (ICU-AIs) is significantly high, i.e., around 51.4%; moreover, the prevalence of this type of infection is estimated to be 9 to 37% in Europe and the USA [5–7]. In addition, in Suetens et al. (2018), the prevalence of ICU-acquired infections was around 20.6% [8]. The most important types of ICU-AIs are ventilator-associated events/ respiratory infections/pneumonia (VAE/VAP), central line-associated bloodstream infections/ septicemia (CLABSI), and catheter-related urinary tract infection (CAUTI) [6]. Ventilator-associated pneumonia accounts for 15% of ICU-AIs [6]. In addition, according to the study by Braga et al. (2018), the most common ICU-acquired infections are VAP and BSIs (53 and 27.6%, respectively) [4].

Recent studies in Iran have shown that the prevalence of HAIs is 1.3 to 10% [9, 10]. Various factors such as the severity of the clinical condition, use of invasive procedures and the type of devices (endotracheal tubes, mechanical ventilation, surgery, central venous catheters, and urinary catheters) play an essential role in increasing the prevalence of HAIs [11, 12].

Hence, HAIs prevention is now a global priority, and the accurate recognition of the magnitude and incidence of HAIs at the national or supranational level using reliable data has become priority for adopting proper infection control measures and reducing the incidence of infection in hospitals, especially in ICU wards. Furthermore, many studies have been performed in Iran at the local and hospital level, but so far, there is no report on the rate of infection at the national level, taking into account all types of infections and by the hospital performance indicators in the ICU wards. Therefore, this study aimed at investigating the rate of ICU-AIs in Iran.

## Methods

### Data sources

#### Iranian nosocomial infections surveillance (INIS)

The national nosocomial infections surveillance, using the definitions provided by the national hospital-acquired infections surveillance system (NNIS), registers the different type of infections, including: ventilator-associated events (VAE), catheter-associated urinary tract infections (CAUTI), surgical site infections (SSI), central line-associated bloodstream infections (CLABSI), pneumonia events & lower respiratory tract infection (PNEU & LRI), infection in bone and joint (BJ), central nervous system (CNS), chorionic villus sampling (CVS), eye, ear, nose, throat or mouth (EENT), gastrointestinal system (GI), reproductive system (REPR), and skin and soft tissue (SST). INIS collects data from about 863 hospitals. It records different variables on HAIs in addition to indicators for each ward and hospital including province, university, affiliated organization, age, gender, type of HAIs, number of hospitalizations, patient-day, number of deaths, number of surgeries, date of infection, and devices used (different types of catheters and ventilator) [13]. In general, INIS data covers more than 85% of Iranian hospitals. The lowest coverage is related to army hospitals, which is 71.87%. INIS data cover 80.23, 85.16%, and more than 85% of private hospitals, governmental hospitals and those managed by other organizations, respectively.

### Hospital statistics and information system (AVAB)

We also extracted performance indicators, including the average LOS, occupancy rate, and bed turnover from AVAB, a system for monitoring and evaluation of different hospitals which facilitate decision policy making. This system registers the hospital characteristics including province, university, accreditation degree, affiliated organization, type of specialty, activity status, ward type, personnel-to-*bed* ratio, death to bedridden ratio*, doctor*-to-*bed* ratio, nurse-to-*bed* ratio, number of death, number of surgery and performance indicators including the average hospital length of stay, bed occupancy and bed turnover in about 1007 hospitals in Iran in 2018 [13].

### Expanded data and missing data imputation

In this multi-center study, INIS and AVAB used to collect data (including 12,586 records in terms of “hospital-ward-month”) on patients with HAIs from 662 hospitals in 2018. The data expanded based on 12 months of the year (13,632 records in terms of “hospital-ward-month”), and then, the last observation carried forward (LOCF) method used to replace the missing data. In LOCF imputation, first, the data on each variable in each hospital ward sorted in terms of months, and the last observation replaced through the forward method; then, the data on the same variable sorted in a reverse order of months. Then, the previous observation replaced for the missing values through the backward method. Finally, the missing value is replace with the average values ​​obtained in the two steps of forward and backward methods. The hospitals with no data on the number of infections and hospitalizations (in none of the months under the study) were excluded.

### Linkage of data sources

In order to link different data sources, we first aggregated the INIS data by summing up the number of patients, number of hospitalizations, and patient-days in each hospital and by the type of ward and the month of diagnosis. In addition, AVAB data was collected as an aggregate data including different variables for each hospital. Then, the two data sources were linked together using a unique code based on the initials of the name of each hospital and the name of the University of Medical Sciences to which the following hospital belonged, using the merge command and as one-to-one key variables by Stata software. Finally, the rate of ICU-AIs calculated using 10,836 records obtained from 579 hospitals.

### The national standard for hospital performance indicators

Hospital performance indicators were categorized based on the standards of the Ministry of Health and Medical Education (MOHME) of Iran as follows:

**Table:** 

Indicators	Desirable	Moderate	Undesirable
**Average LOS (day)**	< 3.5	3.5–4	> 4
**Bed occupancy rate (%)**	> 70	60–70	< 60
**Bed turnover rate**	> 24	17–24	< 17
**Death to bedridden ratio (%)**	< 2	2–3	> 3

Pabon Lasso model is another indicator of hospital performance. Two indices can be used to calculate this model; bed turnover (BTO) and bed occupancy rate (BOR). In this study, the national average of BTO (134.24) and BOR (62.73) was used to plot the Pabon Lasso graphical chart. The plotted Pabon Lasso graph distinguish four zones (zone I, II, III, and IV), where zone I is the inefficient area; hospitals with low BTO and low BOR indicating a surplus of hospital beds relative to the existing demand, zone II is hospitals with high BTO and low BOR (unnecessary hospitalizations, and oversupply of beds), zone III is the efficient area; hospitals with high BTO and high BOR, and zone IV is hospitals with low BTO and high BOR.

### Data analysis

Mean, and median (standard deviation and interquartile range) used to describe age and hospitalization length until infection and hospital LOS. The rates of HAIs also calculated as follows:

#### Rate per patient and patient-days

By each variable, the rate per patient calculated by dividing the total number of patients with ICU-AIs over the total number of hospitalizations, multiplied by 1000. In addition, the rate per patient-days calculated by dividing the total number of patients with ICU-AIs over the total number of patient-days, multiplied by 1000.

#### Rate per device-days

The rate per device-days by each infection type, calculated by dividing the ICU-AIs over the total device-days, multiplied by 1000. For SSIs, the SSIs were divided by the all surgeries in each hospital. The device-associated infections rate was calculated by dividing the ICU-AIs related to ventilator and catheters over the total device-days, multiplied by 1000 [13]. Data were analyzed using Stata software (version 14).

All procedures performed in the study were approved by the ethical committee of the National Institute for Medical Research Development and all methods were carried out in accordance with relevant guidelines and regulations.

## Results

The mean age of the 52,276 patients with HAIs was 47.37 (SD = 30.78) years. The median of hospitalization-infection length and hospital LOS in the patients were 8.28 (IQR = 12.8) days and 21 (IQR = 62.67) days, respectively. Based on the data collected from the 579 hospitals in Iran, the overall rate of ICU-AIs was 96.61 per 1000 patients and 16.82 per 1000 patient-days.

### HAIs rate by ward type and type of infection

**Fig. 1:**
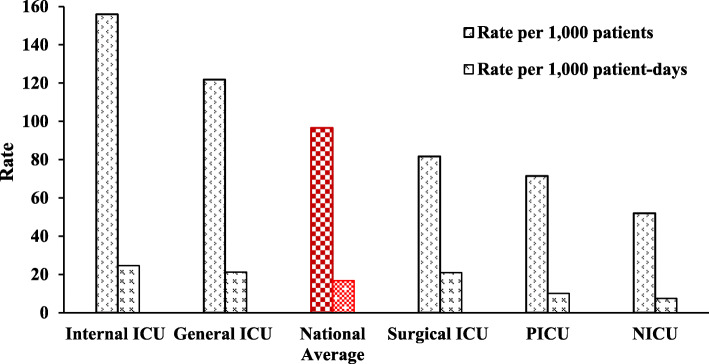
ICU-acquired infections rate per 1000 patients and 1000 patient-days by type of ICU in Iran hospitals-2018

**Fig. 2:**
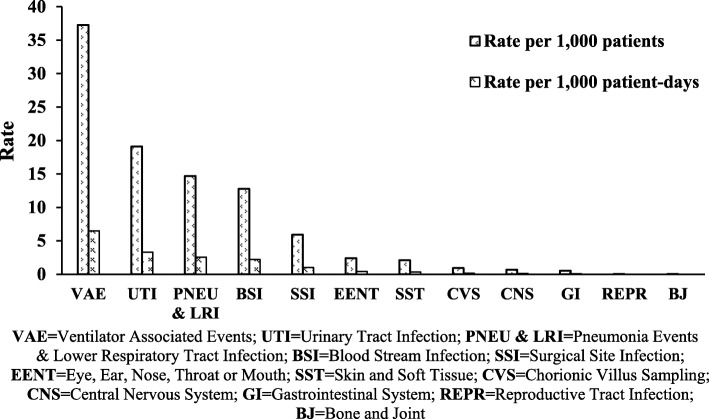
ICU-acquired infections rate per 1000 patients and 1000 patient-days by type of infection in Iran hospitals-2018

**Fig. 3:**
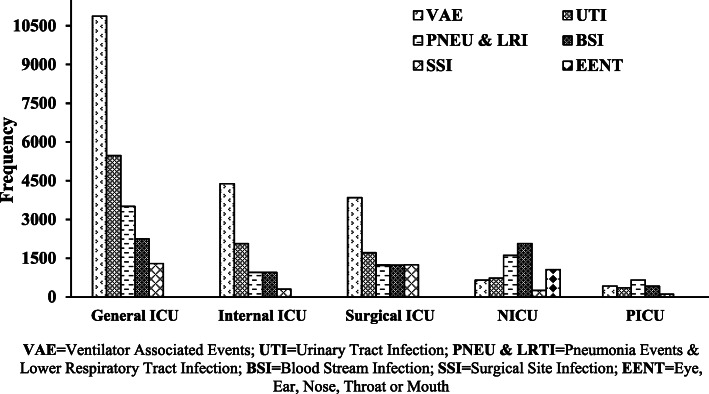
Frequency of main ICU-acquired infections by type of ICU in Iran hospitals-2018

The highest rate of ICU-AIs per 1000 patients and patient-days observed in internal and general ICUs, and the rate of infection in these two wards was higher than the average of national rate **(**Fig. 1**)**. The most common ICU-AIs were VAE (38.59%; 37.25 per 1000 patients, and 6.49 per 1000 patient-days), UTI (19.76%; 19.09 per 1000 patients, and 3.32 per 1000 patient-days), PNEU & LRI (15.21%; 14.69 per 1000 patients, and 2.55 per 1000 patient-days), and BSI (13.23%; 12.78 per 1000 patients, and 2.22 per 1000 patient-days), respectively **(**Fig. 2**)**. The three main HAIs in the general ICUs were VAE, UTI, and PNEU & LRI infections. The three main HAIs in the internal and surgical ICUs were VAE, UTI, and BSI/SSI infections. In addition, the most prevalent HAIs were BSI, PNEU & LRI, and EENT infections in the NICU and PNEU & LRI, VAE, and BSI infections in the PICU **(**Fig. 3**)**.

### HAIs rate by device-days and device-associated infections

**Fig. 4:**
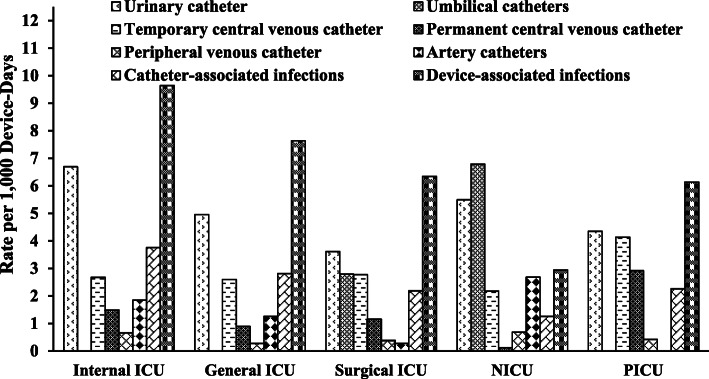
Device-associated infections rate per 1000 device-days by type of ICU in Iran hospitals-2018

Concerning device-days, the rate of VAE and PNEU & LRI infections was 35.52 per 1000 device-days, and the rate of SSI was 23 per 1000 surgeries **(**Table 1**)**. Device, catheter, and ventilator-associated infections accounted for 60.96, 18.56, and 39.83% of ICU-AIs. The device-associated infections in ICUs were 7 per 1000 device-days, and the ventilator-associated infections rate was 26.29 per 1000 ventilator-days. The most common catheter-associated infections were umbilical and urinary tract infections (5.87 and 4.93 per 1000 device-days, respectively). The main catheter-associated infections in the internal, general, and pediatric ICUs were urinary and temporary central venous catheter-related infections. However, the main catheter-associated infections in the surgical and neonatal ICUs were urinary and umbilical catheter-related infections **(**Fig. 4**)**. In addition, the rate of ventilator-associated infections in the internal, surgical, general, pediatric, and neonatal ICUs were 30.41, 28.5, 27.39, 21.56, and 11.7 per 1000 ventilator-days, respectively.

**Table 1:** ICU-acquired infections rate per 1000 device-days by infection type in Iran hospitals-2018

Type of Infection	Num	Device-days	Rate per 1000 device-days
**Ventilator Associated Events, Pneumonia Events & Lower Respiratory Tract Infection (VAE, PNEU & LRI)**	28,131	791896^*^	35.52
**Surgical Site Infection (SSI)**	3202	139216^**^	23^**$**^
**Blood Stream Infection (BSI)**	6920	734,967^**#**^	9.41
**Urinary Tract Infection (UTI)**	10,333	1,413,823^**##**^	7.3

^*^Ventilator-days; ^**^Number of surgeries; ^**$**^ **per 1000 surgeries**

^**#**^**Central venous catheter (Permanent & Temporally)-days;** ^**##**^**Urinary catheter-days**

### HAIs rate by hospital type and affiliation

The rate of ICU-AIs in educational hospitals was higher than in non-educational hospitals (115.45 and 18.42 per 1000 patients and patient-days vs. 79.96 and 14.94 per 1000 patients and patient-days, respectively). The highest rate of ICU-AIs per 1000 patient-days by hospital affiliation observed in semi-government hospitals (19.22 per 1000 patient-days) **(**Additional file 1: Appendix 1).

### HAIs rate by hospital performance indicators

**Fig. 5:**
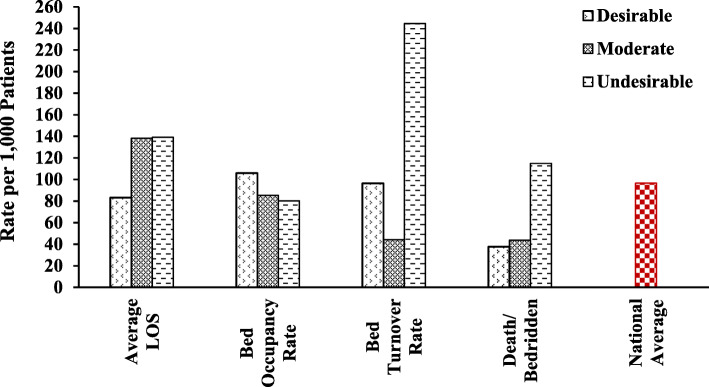
ICU-acquired infections rate per 1000 patients by hospital performance indicators in Iran hospitals-2018

**Fig. 6:**
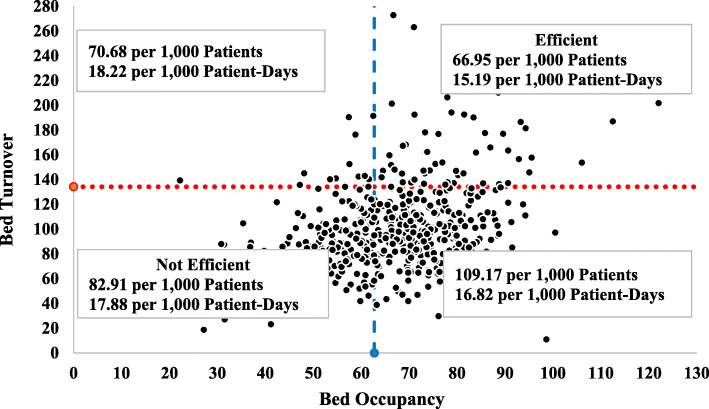
ICU-acquired infections rate per 1000 patients and 1000 patient-days by Pabon Lasso in Iran hospitals-2018

Considering performance indicators including average LOS, bed occupancy, bed turnover, and death to bedridden ratio, the rates of ICU-AIs per 1000 patients were higher in hospitals with an undesirable condition **(**Fig. 5**)**. In addition, the rates of ICU-AIs per 1000 patient-days by all performance indicators in hospitals with the undesirable condition were higher than the national average rate **(**Additional file 1: Appendix 2). On the other hand, based on the results of the Pabon Lasso model, the lowest patients per 1000 and patient-days of ICU-AIs were observed in zone III (66.95 and 15.19, respectively) where there were high bed turnover and high bed occupancy rate/efficient **(**Fig. 6**)**.

### HAIs rate by provinces of Iran

Considering the geographic distribution of ICU-AIs, the highest rates of ICU-AIs per 1000 patients observed in Zanjan, Qazvin, Bushehr, and Hormozgan provinces. Also, the rate of HAIs in 16 provinces was higher than the national average rate. Moreover, the highest rates of ICU-AIs per 1000 patient-days observed in Bushehr, Qazvin, South Khorasan, Hormozgan provinces, and the rate of ICU-AIs 19 provinces were higher than the national average rate. The lowest rates of HAIs per 1000 patient-days observed in Sistan and Baluchestan, Kohgiluyeh & Boyer-Ahmad, and Kerman provinces **(**Additional file 1: Appendix 3).

## Discussion

In the present study, the overall rate of ICU-AIs was 96.61 per 1000 patients and 16.82 per 1000 patient-days (5.03 infections per 1000 patient-days), which were different from the rates observed in Korea (2.76 infections per 1000 patient-days), Japan (2.76 infections per 1000 patient-days), and other countries [4, 14, 15].

In addition, the most common ICU-AIs in this study were VAE (38.59%), UTI (19.76%), PNEU & LRI (15.21%), and BSI (13.23%), respectively. However, in studies by Suetens et al. (2018) and Bianco et al. (2018) in Europe, UTIs accounted for about 20% of all HAIs in the ICUs [8, 16]. Furthermore, in report by Despotovic et al. (2020), UTIs accounted for larger than a third of HAIs observed in adults admitted to ICUs [12]. Nevertheless, in study by Braga et al. (2018) in Brazil, the most common ICU-AIs were pneumonia and BSIs (53 and 27.6%, respectively) that are consistent with our results [4]. Studies in India and China have shown that VAP/VAE in intensive care units were 9.4 and 20.8 cases per 1000 ventilator-days, respectively [17, 18].

All invasive devices are associated with an increased risk of ICU-AIs. In this study, device-, catheter-, and ventilator-associated infections accounted for 60.96, 18.56, and 39.83% of ICU-AIs, respectively, though other studies have reported lower rates [19, 20]. In developing countries, the rates of VAP, BSI, and UTI infections have been reported to be 22.9, 11.3, and 9.8 per 1000 device-days, respectively [21], which are higher than the rates observed in our study.

For the purpose of this study, we had access to only one year of national data and therefore we were not able to investigate trends. Although, the current data is the most available registered data which represent the national situation in Iran, its coverage is about 87.5%. It was not possible to compare not covered hospitals with the included ones according to performance indicators. A random selection of all included hospitals provide a national view of ICU-AI in Iran. Third, the diagnosis of HAIs in countries like Iran is suffering from false negative reports and missing data. Therefore, in this study, we limited our studies to ICU-AIs data which is supposed to be more reliable. The missing data in our analyses managed properly. The present study was the first comprehensive and national study conducted on the rate of ICU-AIs, using both patient-days and device-days, which provide more accurate picture of ICU-AIs.

## Conclusions

The results showed that HAIs are common in the internal ICU wards, while VAE and ventilator-related infections are more prevalent in Iranian hospitals. Therefore, to reduce ICU-AIs, the specific and trained personnel must be responsible for the use of the devices (catheter use and ventilators), avoid catheterization when possible, and remove catheters earlier. Regular training programs, using standard based on infection control guidelines, must be considered for all personnel involved in providing health care for admitted patients.

## Supplementary Information

**Additional file 1: Appendix 1.** ICU-acquired infections rate per 1000 patients and 1000 patient-days by hospital affiliation in Iran hospitals-2018. **Appendix 2.** ICU-acquired infections rate per 1000 patient-days by hospital performance indicators in Iran hospitals-2018. **Appendix 3.** ICU-acquired infections rate per 1000 patients and 1000 patient-days by the province in Iran hospitals-2018.

## Data Availability

The datasets used and/or analysed during the current study are available from the corresponding author on reasonable request.

## Abbreviations

AVAB: Hospital statistics and information system
CAUTI: Catheter-related urinary tract infection
CLABSI: Central line-associated bloodstream infections / septicemia
HAIs: Hospital-acquired infections
ICU-AIs: ICU-acquired infections
ICUs: Intensive care units
INIS: Iranian Nosocomial Infections Surveillance
IQR: Interquartile range
MOHME: Ministry of Health and Medical Education
NNIS: National hospital-acquired infections surveillance system
LOCF: Last observation carried forward
LOS: Length of stay
VAE/VAP: Ventilator-associated events / respiratory infections / pneumonia
