# Supplementary material for: The national rate of intensive care units-acquired infections, one-year retrospective study in Iran
Source: BMC Public Health. 2021 Mar 29;21:609. doi: 10.1186/s12889-021-10639-6 (PMC8006501; doi:10.1186/s12889-021-10639-6)
Supplement: Supplementary file 1 — Additional file 1: Appendix 1. ICU-acquired infections rate per 1000 patients and 1000 patient-days by hospital affiliation in Iran hospitals-2018. Appendix 2. ICU-acquired infections rate per 1000 patient-days by hospital performance indicators in Iran hospitals-2018. Appendix 3. ICU-acquired infections rate per 1000 patients and 1000 patient-days by the province in Iran hospitals-2018. [file 12889_2021_10639_MOESM1_ESM.docx]

**The national rate of intensive care units-acquired infections, one-year retrospective study in Iran**

**Neda Izadi^1^, Babak Eshrati^2^, Yadollah Mehrabi^3^, Korosh Etemad^4^, Seyed-Saeed Hashemi-Nazari^5*^**

^1^Ph.D Student, Department of Epidemiology, School of Public Health and Safety, Shahid Beheshti University of Medical Sciences, Tehran, Iran

^2^Ph.D in Epidemiology, Department of Social Medicine, School of Medicine, Iran University of Medical Sciences, Tehran, Iran

^3^Ph.D in Biostatistics, Department of Epidemiology, School of Public Health and Safety, Shahid Beheshti University of Medical Sciences, Tehran, Iran

^4^Ph.D in Epidemiology, Department of Epidemiology, School of Public Health and Safety, Shahid Beheshti University of Medical Sciences, Tehran, Iran

^5^Ph.D in Epidemiology, Prevention of Cardiovascular Disease Research Center, Department of Epidemiology, School of Public Health and Safety, Shahid Beheshti University of Medical Sciences, Tehran, Iran

**^*^Corresponding Author:** Shahid Beheshti University of Medical Sciences, School of Public Health and Safety, Department of Epidemiology

**E-mail:** [saeedh_1999@yahoo.com](mailto:saeedh_1999@yahoo.com)

**Appendix 1.**

**ICU-acquired infections rate per 1,000 patients and 1,000 patient-days by hospital affiliation in Iran hospitals-2018**

**Appendix 2.**

**ICU-acquired infections rate per 1,000 patient-days by hospital performance indicators in Iran hospitals-2018**

**Appendix 3.**

**ICU-acquired infections rate per 1,000 patients and 1,000 patient-days by the province in Iran hospitals-2018**
